# Supporting self‐management after traumatic brain injury: Codesign and evaluation of a new intervention across a trauma pathway

**DOI:** 10.1111/hex.12898

**Published:** 2019-04-29

**Authors:** Petra Mäkelä, Fiona Jones, Maria Inês de Sousa de Abreu, Lucinda Hollinshead, John Ling

**Affiliations:** ^1^ King’s College Hospital London UK; ^2^ Faculty of Health, Social Care and Education, Kingston University and St George’s University of London London UK; ^3^ Bridges Self‐Management Limited London UK; ^4^ Present address: University of Westminster London UK; ^5^ Present address: London School of Hygiene & Tropical Medicine London UK; ^6^ Present address: St. George’s University Hospitals NHS Foundation Trust London UK

**Keywords:** codesign, hospital care, patient‐centred care, quality improvement, self‐management support, teamwork, traumatic brain injuries

## Abstract

**Background:**

Supported self‐management (SSM) is a recognized approach for people with long‐term conditions but, despite the prevalence of unmet needs, little is known about its role for people with traumatic brain injury (TBI).

**Objectives:**

To codesign an SSM intervention with people with TBI and evaluate feasibility of implementation through multidisciplinary staff across a trauma pathway.

**Setting and participants:**

People who had previously been admitted to a Major Trauma Centre following TBI and family members participated in a series of codesign activities. Staff attended SSM workshops and used the intervention with patients in acute and rehabilitation settings.

**Methods:**

We used Normalization Process Theory constructs to guide and interpret implementation. Knowledge, beliefs and confidence of staff in SSM were assessed through pre‐ and post‐training questionnaires, and staff, patients' and families' experiences were explored through semi‐structured interviews. Qualitative data were analysed thematically, and clinical measures were mapped against a matched sample.

**Results:**

Codesigned resources were created and used within an SSM approach for which 110 staff participated in training. Evaluation demonstrated significant differences in staff SSM confidence and skills, following training. Qualitative evaluation revealed adoption by staff, and patients' and families' experiences of using the resources. Challenges included reaching staff across complex pathways to achieve collective implementation.

**Conclusion:**

This is the first project to demonstrate feasibility of SSM for people after TBI starting in an acute trauma setting. Through an open approach to codesign with a marginalized group, the SSM resources were valued by them and held meaning and relevance for staff.

## 1 BACKGROUND

Between 1.0 and 1.4 million people attend hospital in the UK annually with a head injury,^1^ and around one fifth require admission to hospital. Traumatic brain injury (TBI), defined as an alteration in brain function or other brain pathology caused by an external force,^2^ is a leading cause of disability in working‐age adults.^3^ Good physical recovery usually allows discharge directly home from the acute setting, with referral to inpatient rehabilitation services for a minority.^4^ Though an injury may be clinically categorized as “mild,” individuals can go on to experience longer‐term cognitive, psychological, emotional and social effects, frequently resulting in “hidden disability”.^5^ Families navigate a complex, changing situation that may include mood disturbances associated with their relative's injury, shifts in family relationships and changes in financial resources.^6^ People who are discharged from hospital after TBI are often referred to as “walking wounded,” a label which can diminish the broad impacts and need for adaptation to challenges in everyday life.^7^

National Institute for Health and Care Excellence (NICE) guidance in England^8^ recommends that, on discharge from hospital following head injury, patients should be provided with an information sheet. Information giving has limited effectiveness in other conditions, such as stroke.^9^ However, for many people, this method represents the extent of support received as they attempt to reintegrate into everyday life. Health‐care services often respond reactively to emerging consequences of TBI and, in the context of complex referral routes and care pathways, people with TBI may not be offered follow‐up, particularly if this was not considered to be their “primary diagnosis”.^10^ In the absence of support beyond the acute event, direct medical costs accrue when people seek support through general practitioners, emergency services and referrals to a range of specialty clinics,^11^ though the assessment of the economic burden of TBI to patients, families and society represents a relatively new area of exploration.^11^

Support for self‐management has become a prominent strand of health‐care policy for long‐term conditions.^12^ In the National Health Service (NHS) in England, this is considered a core part of transformation as set out in the “Five Year Forward View”.^13^ Frameworks for SSM encompass a range of strategies at levels of individual, health‐care professional, organization and systems. However, when underpinned by neoliberal philosophy of individual self‐governance, this policy focus may seem to place preference on individual responsibility for managing a condition.^14^ This idea is supported by Ellis et al in their work on conceptualizations of the “good self‐manager,” describing someone who uses services “appropriately,” uses knowledge to manage risks and actively applies information to make decisions.^15^ This focus also aligns with the move towards measurement of “patient activation,” where those deemed “more activated” are considered to have greater self‐management capability.^16^ These concepts risk exacerbation of disparities in access to support, due to judgements made by health‐care professionals about which patients are “activated” and likely to benefit.^17^ Broadening of considerations beyond clinicians' priorities is required, if aspects of support which people value most are to be included and socially distributed resources are to be recognized.

The conceptualization of TBI as an abrupt‐onset, acute condition can hinder understandings of longer‐term challenges. Unlike many other long‐term conditions, self‐management as a framework for support is rarely considered for people after TBI. The focus is often on physical activity^18^ or the delivery of education about brain injury.^19^ Widening access to SSM beyond “all or nothing” delivery is a challenge that remains unaddressed, especially when considering complexities such as cognitive impairment.

More than a decade ago, a King's Fund report recommended that organizations should develop flexible approaches to SSM, highlighting a need for development of professionals' skills in this approach.^20^ However, research suggests that efforts to promote support for self‐management have rarely achieved the sustainable improvements that policy leaders anticipate.^21^ Achieving SSM in everyday practice increasingly needs to recognize organizational contexts and values, as well as motivations and behaviours of health‐care professionals. Challenges for SSM interventions include commonly encountered objections from health‐care professionals about involving people in their care, for example: “We already do it,” “Patients don't want it,” “It's not appropriate,” or “There isn't enough time to do it”.^22^ ^,p.33^ Recognizing such perspectives, we sought to collaborate with patients, families and staff, who had experience of health care after TBI, to codesign an SSM intervention which would be responsive to the complex, acute contexts of intended implementation.

## 2 AIM AND OBJECTIVES

The overall aim of this improvement project was to develop a shared multidisciplinary approach for staff to support people after TBI and their families from the acute injury onwards. Our objectives were to (a) develop a new SSM intervention through a staged process of participatory codesign; (b) deliver interactive training in multi‐professional groups from across the traumatic brain injury pathway (acute, rehabilitation and community settings); and (c) to evaluate feasibility of implementation of the new SSM intervention across a trauma pathway.

## 3 METHODS

The principle of coproduction underpinned our approach, starting with people that support is intended for, exploring what they think works well and what needs to be addressed, thereby contesting the traditional biomedical model and maintenance of control by professionals.^23^ We considered codesign to refer to “patients and carers working in partnership with staff to improve services”.^24^ ^,p.1^ Settings for this improvement project were an NHS organization across two geographical sites (acute and rehabilitation services of a Major Trauma Centre) and a third sector organization supporting people with brain injury in two community day centres.

### 3.1 Ethical approval

According to the policy activities that constitute research at the host organization, this work met criteria for operational improvement activities exempt from ethics review. However, we obtained ethical approval from the Faculty of Health and Social Care Sciences Research Ethics Committee, Kingston University and St George's, University of London, for the improvement activities and the evaluation. All participants provided informed consent. We followed ethical principles for good practice in codesign.^25^

### 3.2 Self‐management support model

The foundation of this project followed an established SSM intervention, which has previously been implemented and evaluated for people following stroke.^12^ The intervention is underpinned by principles of social cognitive theory and the concept of self‐efficacy, which concerns an individual's beliefs in their capabilities to produce given attainments.^26^, ^27^ Self‐management approaches to increase self‐efficacy incorporate goal mastery, learning from the experience of others in a similar situation, psychological or physical feedback, and social persuasion. The established intervention follows seven principles of problem‐solving, reflection, goal setting, accessing resources, self‐discovery, activity and knowledge.^28^ Implementation of this self‐management support is through existing health‐care interactions that are tailored to patients' needs and is achieved through an interdisciplinary approach. We sought to contextualize this existing model to challenges encountered after TBI, through collaboration with patients, family members and service providers.

### 3.3 Codesign approach

We used an iterative and open approach to codesign of the intervention, through a series of four focus groups with 8‐10 people in each. The project was discussed with people with TBI during the course of planned reviews by project team members in the brain injury clinical service. Those expressing interest were later invited to focus groups. Participants had been admitted to a Major Trauma Centre with TBI between several months and several years previously, and were joined by family members or other supporters. The focus group discussions were facilitated by members of the project team, incorporating areas such as challenges, successes and strategies that participants had been finding helpful and wished to share with others going through a similar experience. Focus group participants reviewed copies of the existing book for supporting self‐management after stroke and provided direction about the appearance, content and layout of the TBI books. They gave examples of their experiences to shape content of staff workshops, including illustrations and key occurrences when they felt held back from self‐managing or required different support from staff. Discussions within these groups shaped next steps for the project, as people shared opinions and identified priorities; for example, family members identified the need for a separate resource for families and friends.

In creating the resources, fourteen people living with TBI and family members were interviewed for 1‐2 hours each, guided by topics that had been identified as important through extraction of themes from the focus group transcripts. The individual interviews were fully transcribed, and from these, vignettes were developed for inclusion in the resources, using contributors' own words. A similar process was completed for the “family and friends” book, in which seven families contributed their ways of coping. Following these collaborative activities, prototype books were produced and reviewed by members of codesign groups and by an advisory group of multidisciplinary staff representatives. The final, newly developed intervention comprised three interrelated components: (a) three‐stage training workshops for multi‐professional staff, and abbreviated training for leads, managers and others; (b) a patient‐held book with fourteen vignettes, strategies and space to record personal targets and progress; and (c) a book for family and friends, aiming to share ideas and experiences of parents, siblings, partners and children and to provide ideas about promoting self‐management with their relative or friend with TBI (see Supplementary Online Material Data S1 and S2 for sample pages from each of the codesigned books). In addition, a group of people living with TBI and family members continued to contribute to the direction of this work and later collaborated in the development of a video for staff training purposes.

### 3.4 Implementation

**Figure 1:**
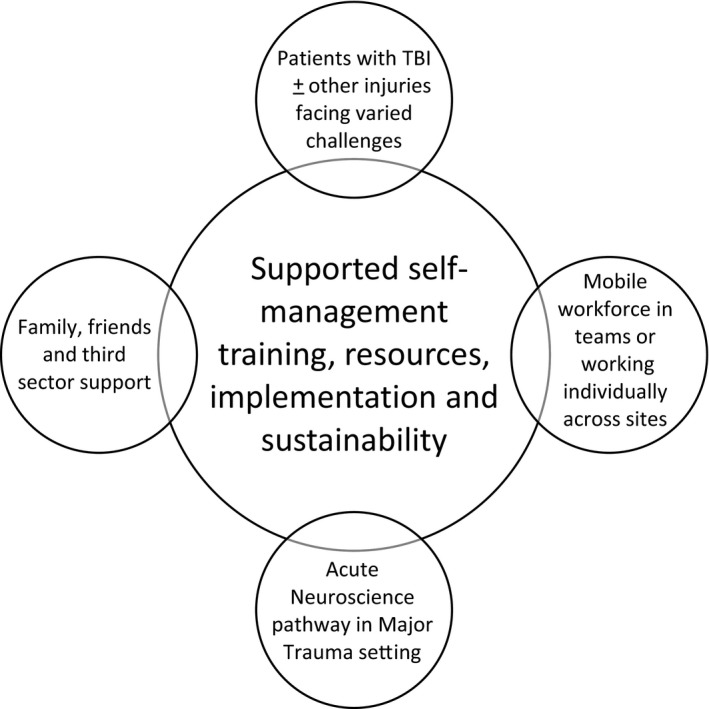
Interacting components of supported self‐management implementation

Implementation of SSM through usual ways of working represents a complex intervention with multiple interacting components (see Figure 1). As a new intervention to support self‐management challenges conventional ways of working, we used components of Normalization Process Theory (NPT) to guide our approach and to evaluate implementation.^29^ NPT describes how practices can become routinely embedded in social contexts, considering components of coherence (“what is the work?”), cognitive participation (“who does the work?”), collective action (“how does the work get done?”) and reflexive monitoring (“how is the work understood and sustained?”).^29^

We sought understanding of contextual factors by taking an iterative view of context as “part of the action” of implementation, which changes over time, rather than a static backdrop.^30^ We considered everyday language and interactions in the organizational setting, with focus on verbal communication and exchanges with stakeholders, through meetings with representatives from therapies, nursing, medical, psychology, managerial and third sector staff, and a group of people living with TBI and family members.

An open invitation was extended to all staff working across the TBI pathway through clinical managers, awareness‐raising sessions, attendance at team meetings by the project team and email correspondence, to generate engagement in the project and to recruit staff for the training. Real‐time feedback to project clinical coordinators, from staff who were integrating the approach into their practice, allowed exploration of responses within dynamic health‐care settings, taking account of attitudes towards the intervention. We were able to use examples from staff that related directly to caring for people with TBI and their families as well as those relevant for the work setting (acute ward, rehabilitation unit or home) to give a sense of local context and practicality to the training. We considered engagement with “actors” (professionals), “objects” (training content, codesigned books) and “environment” (organizational structure, context and processes), aiming to arrive at a stage when SSM could be routinely embedded into usual multidisciplinary practice.

### 3.5 Evaluation

In our approach to evaluation, we recognized that the innovation phase of an intervention requires a different approach to testing phases, as changes in behaviours and interactions between professionals, patients and families may represent steps towards successes, before measurable outcomes are achieved.^30^ This evaluation aimed to assess feasibility of integrating the SSM intervention into usual health‐care processes and explore ways patients and families perceived and used the support.

An explicit programme theory articulates how the intervention is proposed to lead to improved outcomes and can promote transfer of learning from one project to the next.^31^ At the outset of the project, we proposed that the components of the intervention (staff training, patient and family books) would enable staff to support self‐management within usual interactions; staff would use their time more effectively by focusing on a collaborative model of care; and patients and families would experience personally meaningful support in coping after TBI.

We used a mixed‐methods approach with standardized measures for (a) changes in professionals' self‐reported knowledge, beliefs and skills for supporting self‐management, and (b) to evaluate representativeness of the patient sample with whom staff used the SSM approach during implementation, compared with a matched comparator patient group. Patients were matched according to age, gender, whether they had required initial neurosurgical management and their length of stay in the Major Trauma Centre. Clinical measures, already in use within the system of follow‐up for people following TBI, were captured to enable characterization of the intervention group. We used qualitative methods to explore contexts, processes and responses to the intervention (see Table 1).

**Table 1:** Overview of evaluation plan

Processes	Outcomes	Balancing aspects
Numbers and roles of staff attending training Patient numbers, demographics, settings Questionnaires, interviews, focus groups with staff and patients Staff case reflections	Professionals Questionnaires and qualitative interviews: ‐ Attitudes and beliefs ‐ Implementation experiences Patients ‐ Standardized measures ‐ Qualitative interviews	Qualitative evaluation: ‐ Staff perceptions of practicalities including time needed, challenges and benefits ‐ Normalization activities and perceived barriers ‐ Impact perceived by families and friends

### 3.6 Data generation

Field researchers collected data in acute wards of a Major Trauma Centre in the NHS in England, the associated brain injury follow‐up clinic, neurorehabilitation unit and brain injury charity day centres in the community. We developed topic guides for staff, people with TBI and families through review of focus group transcripts and added questions about experiences of using the codesigned resources. We carried out semi‐structured interviews with a range of purposively sampled multidisciplinary staff members across settings, people who had experienced TBI, family members and other supporters.

Patients and family members who had been introduced to the self‐management support intervention during their clinical care, or while attending the brain injury charity day centre, were invited to take part in interviews by the project clinical coordinators. Purposive sampling continued until people with a range of service experiences and social circumstances had been included, within pragmatic considerations according to participant availability. Following informed consent, interviews with patients and families were audio‐recorded and transcribed, and detailed notes were taken of the interviews with staff, including verbatim quotations. Field researchers collected standardized measures and patient questionnaires during outpatient follow‐up, or through telephone contact when preferred by patients and families.

We collected data regarding professionals' knowledge, attitudes and beliefs about SSM through pre‐ and post‐training questionnaires. Participants rated their level of concordance with statements related to self‐management generated from literature.^32^, ^33^ An online staff survey and qualitative data from staff interviews facilitated further understanding of engagement.

Standardized clinical measures were collected during outpatient follow‐up approximately three months after hospital discharge, using the 36‐Item Short Form Health Survey (SF36)^34^; Hospital Anxiety and Depression Scale (HADS)^35^; Rivermead Post‐Concussion Symptom (PCS) Inventory^36^; and General Self‐Efficacy Scale.^37^

### 3.7 Data analysis

We compared staff data pre‐ and post‐training using the Wilcoxon signed rank test. We summarized patient data for comparison with the matched historical group (admitted to the acute trauma service during the previous year). We used inductive thematic analysis for qualitative data, as recommended for preliminary health service research.^38^ Codes were phrases relating to experiences as a recipient of the approach or as a clinician enacting the intervention in everyday practice. We grouped codes to develop categories and themes across the whole data set, by re‐reading transcripts and adjusting themes to reflect new data. This process was carried out by FJ with PM as a peer reviewer, through iterative discussion until no new themes were identified.

## 4 RESULTS

Approximately 70 multidisciplinary staff from acute, rehabilitation and third sector settings attended three‐part training workshops (see Table 2), and 40 staff including clinical leads, managers and peer support volunteers attended an abbreviated session. Questionnaire data are available in the online Supplementary Material Data S1 and S2. The analysis identified significant changes in self‐reported knowledge, beliefs and skills in supporting self‐management after TBI, following training. Changes demonstrated a shift from didactic approaches, such as provision of information and staff determined goal setting, towards the collaboration that underpins the SSM intervention. In addition, after training, significantly fewer staff felt time would need to be set aside to support self‐management.

**Table 2:** Professional backgrounds of staff attending training workshops (data available for 62 attendees)

Role	Number
Nurse	18
Occupational Therapist	10
Physiotherapist	7
Rehabilitation Assistant	6
Third Sector (Headway)	5
Health‐care Assistant	3
Psychologist	3
Doctor	2
Speech and Language Therapist	1
Other	7
Total	62

**Figure 2:**
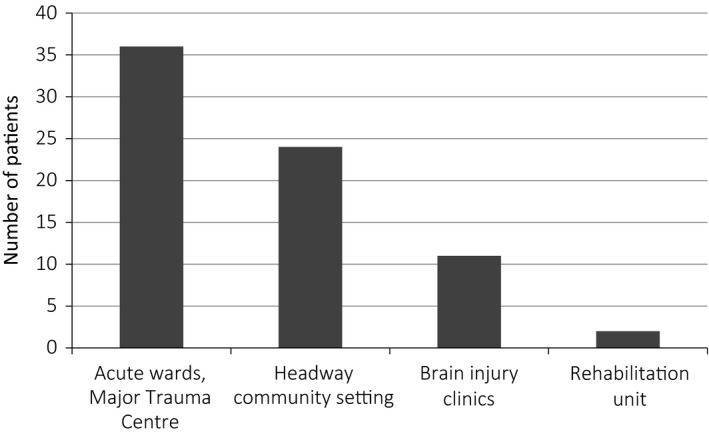
Settings of implementation (n = 73)

**Figure 3:**
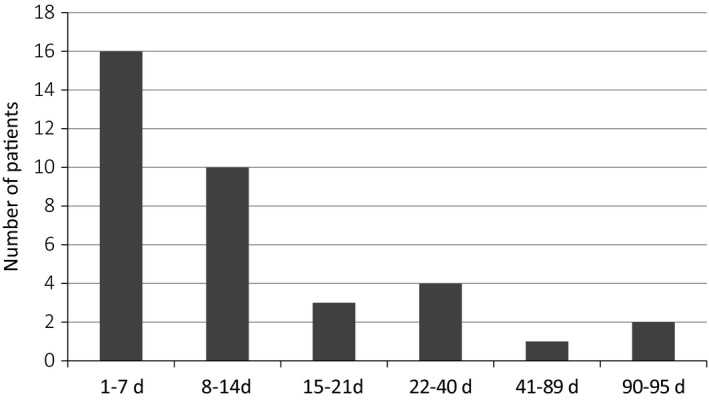
Introduction to intervention: time after acute admission (n = 36)

Implementation and evaluation processes took place over a five‐month period in 2015. We collated quantitative data for a sample of 73 patients who had experienced TBI and had been introduced to the SSM intervention. Fifteen patients and family members took part in qualitative interviews. Demographic and injury‐related data are available in the online Supplementary Material Data S1 and S2. The group with whom staff had implemented the SSM approach were broadly representative of the range of patients admitted to the Major Trauma Centre, comprising 67% male, 48% white British and ages between 16 and 80 years. Implementation within the acute setting exceeded that in later pathway stages (shown in Figure 2), consistent with our intention to provide support early after admission. The majority of patients and families were introduced to the intervention between 1 and 2 weeks after injury (see Figure 3). The range of injury severities, represented by need for acute neurosurgical intervention and length of acute stay, is shown in the online Supplementary Material (Appendix S3).

Collection of standardized follow‐up measures was limited due to non‐attendance at clinics, appointments outside the project interval and difficulties contacting patients. Data for HADS and SF36 were available for a subgroup of 18 patients who received the intervention in the acute setting, and were matched to the historical comparators (see Supplementary Data, Appendix S4). Data suggested higher HADs for the matched historical sample, consistent with greater levels of anxiety and depression in the pre‐intervention sample. For SF‐36, mean scores for physical health for both groups were in the “well below average” range; the mental health mean score for the historical group was in the “well below average” range and “below average” for the intervention group. Self‐efficacy scores for the intervention group are shown in online Supplementary Data (Appendix S4); comparator data were unavailable as this score was not in use for the matched sample. Scores for the Rivermead PCS Inventory were documented for too few patients historically to enable comparison.

### 4.1 Qualitative findings

The main themes identified in staff interviews were “common language and understanding”; “stories and small steps”; “who is ready and when to use”; and “changes to practice.” In addition, three main themes were generated from patient and family interviews, relating to the role of the intervention in “helping acceptance”; “feeling less alone”; and as a prompt to “remember targets and plans.” We illustrate these themes with quotes from staff, patients and families.

#### 4.1.1 Staff

##### Common language and understanding

Staff reflections revealed ways they supported self‐management through subtle changes in language and approaches to supporting people with TBI. In some cases, the changes became integrated into usual ways of working.

I think that actually once you start working in this way, it becomes self‐sustaining because you get into a pattern of working where I have seen positive changes and positive results, so I have carried on doing it… it is a natural thing to do once you get into the habit. (Therapist, Rehabilitation ward)

Such changes also became incorporated within team communication methods, potentially providing a sustaining mechanism:

We now have a consistent approach [which has] assisted us in increasing our MDT working when discussing implementation…it also means the language and approach is the same. Our approach is now more patient focused rather than professional driven. (Therapist, Acute Trauma)

##### Stories and small steps

The codesigned books were appreciated by staff as a useful tool to support self‐management. Accounts illustrated the impact of using different strategies and ways to include family members*:*

All she could see was how far away she was [from returning to work], and not how to get there. So it was very much a case of breaking it all down …she responded well to the idea that these were little steps towards her bigger goals. (Nurse, Acute Trauma)

With the family book this also gives us another dynamic and we now have a tool to educate family members when their loved one is going through a very distressing phase. (Therapist, Acute Trauma)

##### Who is ready and when to use

Staff training sessions often generated discussions about the readiness of patients for self‐management, particularly in the acute setting. Distinctly different views and experiences were expressed by staff, for example:

She had found [the book] very helpful because she couldn’t sleep, and then looked back later on what she had written down during that time and was amazed at what she had managed to write there. This would have been around one week after her brain injury, here in one of the Neurosurgical wards. (Nurse, Neurosurgery)

My primary difficulty with implementing [the SSM intervention] is that within the acute phase of TBI the majority of patients whether they are mild or moderate are not yet ready to access it. (Therapist, Acute Trauma)

The different experiences reveal problems associated with a focus by staff on “the book” as the intervention, leading to preconceptions of SSM as a challenge for some patients, but also illustrate how some staff proceed with the approach without questioning whether it is the “right time.”

##### Changes to practice

Post‐implementation reflections revealed shifts in practice that were consistent with findings from questionnaire data assessing staff attitudes and beliefs. The following quotes illustrate how clinicians acknowledged that their existing attitudes may not be conducive to self‐management:

I think that there is an element where we want to rescue people. Having been on the [SSM] training that is definitely something that has changed for me. I push things back into people’s own courts a lot more. (Therapist, Rehabilitation ward)

I need to spend more time in asking before prescribing, and having a peer discussion with patient and family, rather than speaking only on clinical matters. (Medic, Major Trauma Centre)

Staff adopting the intervention demonstrated openness when talking about their practice, critical reflection on their interactions with patients and families, and a willingness to “actually give it a go.”

#### 4.1.2 Patients and families

##### Helping acceptance and understanding ups and downs

A number of patients described ways the SSM intervention helped them to recognize a path moving forward in their recovery:

This book makes me comfortable because when I am reading it is just me and the book…The stories are good; they make me feel I don’t have to hide anything. The more I read how [contributors] had head injury, the more I can open up. (Person with TBI, Neurosurgical ward)

Family members expressed some mixed views, such as differing perspectives on how useful resources were for their relative compared to themselves:

I could see the difficulties [my husband] was having and similarities with some people’s stories in there but he thought he was better, himself. (Wife of person with TBI, Community)

I found the ‘Changes in your family member/friend’ most interesting. Can see that now, everything is not 100%. Without you reading, it would all come as a shock. (Father of person with TBI, Neurosurgical ward)

##### Feeling less alone

Patients also talked about using the book to understand their challenges and find ways to cope. They took comfort in reading other people's own words, alongside their own unfamiliar experiences and challenges encountered over time:

I tend to look at other peoples’ experiences, see how they resolve their issues and try and transfer it to my situation. (Person with TBI, Community)

Families also appreciated reading about how other families had coped:

You think that it is just you, so it is nice to hear about other families’ experiences. Without those stories, I would not have thought about other…it puts it into perspective. (Wife of person with TBI, Community)

##### Help to remember targets and plans

People followed ideas from contributors in the book to help them make a plan, reflect on progress and set targets, illustrating a key strategy promoted in the SSM intervention:

I write bullet points down now and it opens the door for more conversation with people. So, that is a great point from someone that never used to take notes, or was that way inclined. (Person with TBI, Community)

In summary, discussions with staff showed ways the SSM intervention was perceived to differ from usual practice on an individual level and, in pathway settings where teamwork was already established for people with TBI, their cognitive participation became apparent through accounts of incorporation into usual team practices. However, strategies to achieve collective action by staff across settings (eg, when transferring care to another ward) were not demonstrated within the time frame of this project. Family members' accounts, including their reflections on the period of the acute admission, demonstrated their own cognitive participation by use of resources according to their relative's changing situation over time, a process which continued after discharge. In this way, interactions with staff members using the SSM approach led to unforeseeable shifts in patients' and families' ways of collectively managing changes and challenges that subsequently unfolded.

## 5 DISCUSSION

Understandings of coproduction vary, and what is being produced is not always apparent.^39^, ^40^ This study addresses an identified gap, providing a tangible example of codesign within an acute trauma setting, where biomedical concerns traditionally dominate. Through an inclusive approach, we gained closeness to the complexity of experiences and understanding of local needs within a major trauma pathway. Attention to families' contributions allowed for expansion of the notion of coproduction of SSM, beyond health‐care professional‐patient interactions. By collaborating with a marginalized group, the resources created held meaning and relevance to them and, in turn, to staff.

The strengths of this project come from learning about implementation across multiple professional groups and contexts, with a cohort of patients previously excluded from self‐management programmes and under‐represented in participatory quality improvement.^41^ Limitations included the duration of evaluation within this project's time frame, in which we were not able to assess sustainability in settings where frequent staff turnover is unavoidable. We discuss findings in more detail below, drawing on headings recommended in SQUIRE 2.0.

### 5.1 Impact on people and systems

We needed to understand beliefs, knowledge and confidence at the individual staff level of evaluation, but we also sought to understand how they made sense of the intervention in day‐to‐day activities and what action they took as a group, addressing “coherence” and “cognitive participation” constructs of NPT. Our previous work has suggested that staff can perceive lack of time, pressures of an acute medicalized environment, and patients with cognitive deficits to present common challenges for integration of SSM,^42^ yet the qualitative evaluation in this study revealed engagement from patients, families and clinicians working with these factors. We also gained accounts of how families had used the codesigned resources and integrated self‐management strategies frequently following discharge, shaping their response to the changing situation and addressing the lack of guidance for people following discharge after TBI and their families. As the subsequent use and value of the intervention within families' lives and ways of coping are unknown to staff at the outset, there is a need for suitable feedback processes to build understanding and facilitate reflexive monitoring by staff using the intervention.

The codesigned books embody a person‐centred approach, which has a “natural fit” with patients and families and provides staff with a tangible, shared mechanism to implement SSM strategies within everyday work. Patients and families gave distinct examples of how they utilized the books' content to aid self‐management but they were not aware of any particular approach by staff, as expected when integrating SSM into everyday interactions. By comparison, clinicians referred to the “practice” of supporting self‐management, with and without the books. This reflects an emphasis on strategies used by clinicians within their clinical interactions, to foster confidence by focusing on the assets and skills of patients and families. Within the SSM training, staff are discouraged from perceiving the intervention as “a book,” particularly when used without interactional support. Nonetheless, examples were identified where clinicians asserted that patients were “not ready” for the intervention, with reference to “giving out the book.” The concept of patients meeting criteria for SSM is not uncommon yet can exclude patients who may have the most to gain. Conflation of “the approach” with “the devices” also demonstrates an established myth of person‐centred care: “It's easy! A tool will do”.^43^ ^,p.383^ Our findings highlight enduring power imbalances, when health‐care professionals decide which people are “right” for an SSM intervention.

### 5.2 Limitations and future directions

Contexts of implementation are critical to understanding how an intervention may be adopted and adapted across different settings or time periods, while retaining key principles.^44^, ^45^ Although social desirability^46^ influences may have affected responses to questionnaires and interviews, we adopt an appreciative inquiry ethos to discover from these data what “gives life” to the intervention within the living system.^47^ Conditions present at the outset of this project shaped the collaborative efforts that were required to gain organizational approval and clinical leadership support for the SSM intervention. A Major Trauma Centre is a relentlessly busy setting in which to attempt behaviour change, encompassing organizational factors such as imbalances in power of different stakeholders, perceptions of incentives to collaborate and history of co‐operative working across professional groups.^48^ Structural factors also impacted on implementation, including the lack of co‐located beds for patients admitted after brain injury. Although presenting challenges in the achievement of a shared approach within this project, such contextual factors also open possibilities for future understandings of how the SSM intervention may be disseminated through internal initiatives.

There was insufficient opportunity to address longer‐term operational issues of collective action in embedding and sustaining the approach within the scope of this project.^49^ However, conflicting attitudes towards SSM may impact specifically on the collective action required for normalization of new ways of working and, within implementation processes, it is critical that challenges for staff are acknowledged. At the individual level, professionals tend to hold differing worldviews in accordance with their training and experience; staff in acute health‐care settings may be unfamiliar with enabling people, and the family and friendship systems around them, to take a more active role in recovery. Supporting self‐management can therefore require a shift in culture, which may be facilitated through authenticity achieved by the codesigned SSM intervention.

Structures and materials through which the intervention can become embedded include language used by staff, goal‐setting practices, multidisciplinary documentation and formats of family meetings. However, reaching a stage of sustained implementation as “the way we do things here,” despite everyday pressures and competing demands, requires further change management. We recognize that mechanisms for sustaining awareness and training within teams are a necessary part of implementation. We have identified a number of approaches, which have subsequently been initiated, including “champions,” masterclasses, refresher training and different modes of learning such as teaching films and web‐based support. Ultimately, professionals need to experience and share understanding of longer‐term effects of a person‐centred approach, to achieve a level of normalization in their practice. Through identification of conditions of context necessary for their success, we can enhance learning from those efforts, to inform our further development of the programme theory.^31^

## 6 CONCLUSION

This is the first project to codesign self‐management support with people after TBI and demonstrate implementation in a trauma pathway. A whole‐systems approach to self‐management, starting early after injury, can help to address hidden needs, achieve earlier impact and change the focus of health‐care interactions. Implementation across professional groups contributes to sustainability and optimizes support throughout the 24 hours of inpatient services. Integration into usual interactions can be more effective than providing a separate intervention, is less costly and promotes shared understanding. This project has confirmed our preferred stance of SSM as a continuum, to reduce gate‐keeping assumptions that patients with TBI are not ready, “activated” or in the right setting. We propose a need to build on knowledge and skills within a local setting, rather rigidly adhere to a notion of intervention “fidelity”.^50^

Major Trauma Centres are now established across England, each linked with supporting Trauma Units. Since this project, further funding has supported spread of the intervention across a Major Trauma System. Our next steps will focus on synthesizing findings to develop a flexible intervention across trauma settings by collaborating with patients, families and staff, and exploring interactions of organizational contexts with normalization of the intervention in practice. We recognize that additional evaluation methods are necessary, particularly to capture economic and social impact at individual, service and organizational levels.

## CONFLICT OF INTEREST

FJ is the founder and director of the social enterprise Bridges self‐management. PM, MdS, LH and JL have no conflicts of interest to declare.

## DATA AVAILABILITY STATEMENT

The data that support the findings of this study are available from the corresponding author upon reasonable request.
