# Supplementary material for: Supporting self‐management after traumatic brain injury: Codesign and evaluation of a new intervention across a trauma pathway
Source: Health Expect. 2019 Apr 29;22(4):632–42. doi: 10.1111/hex.12898 (PMC6737836; doi:10.1111/hex.12898)
Supplement: Supplementary file 1 [file HEX-22-632-s001.pdf]

Appendices: supplementary online material

Appendix 1

Evaluation of changes in staff attitudes, knowledge and beliefs about supporting self-management

The following statements showed a significant change towards concordance with the Bridges supported self-management approach after training (pre- versus post- training, using Wilcoxon signed rank test):

- Educating people with TBI is the most important part of supporting self-management (fewer participants agreed post-training; p=0.004)
- A specific time needs to be set aside to support self-management (fewer participants agreed post-training; p=0.045)
- It is important to guide people with TBI to set 'SMART' goals[1] (fewer participants agreed post-training; p=0.006)
- Practitioners should use their experience and expertise to direct the goal setting process (fewer participants agreed post-training; p=0.001)
- It is important to advise an individual if they have unrealistic hopes about what they can achieve (fewer participants agreed post-training; p=0.004)
- Goals phrased in the person's own words can be more effective (more participants agreed post-training; 0.01).

1. 'SMART' (specific, measurable, achievable, realistic/ relevant and timed [67]) goals are considered inconsistent with the Bridges approach to supporting self-management approach.

Appendix 2

Demographic and injury-related data for patient sample receiving intervention

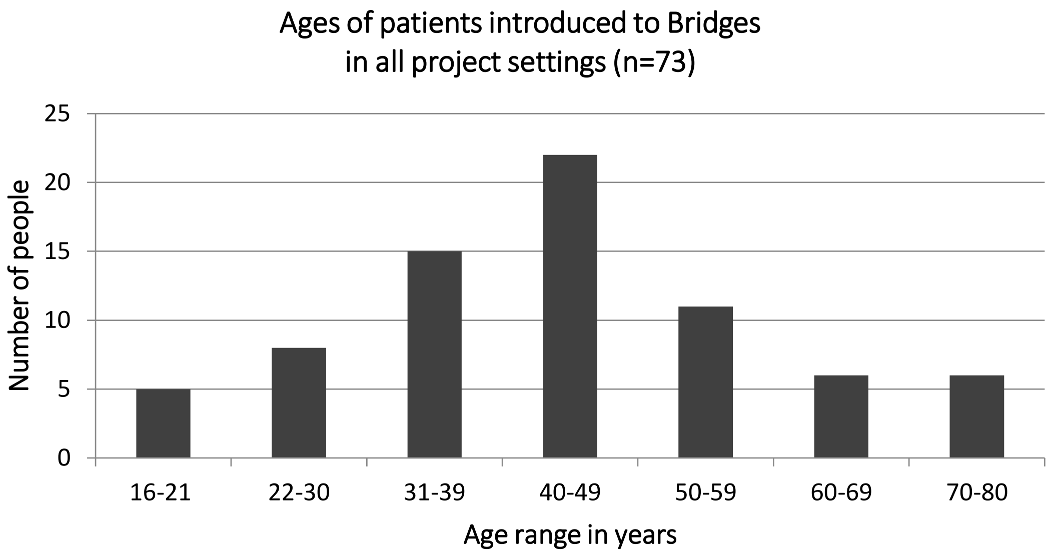

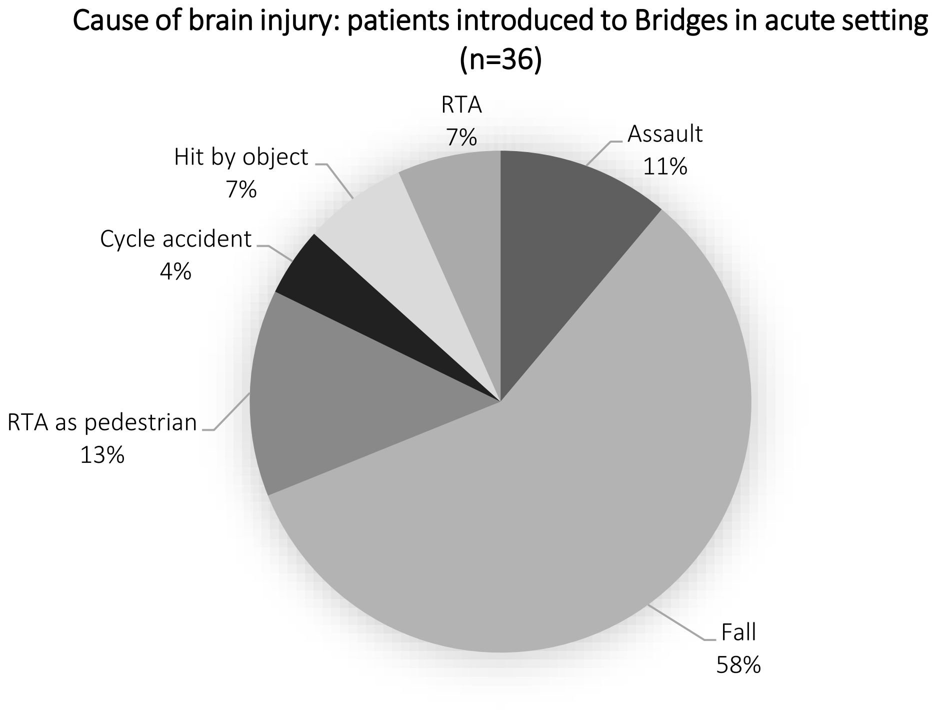

Appendix 3

Markers of injury severity for intervention and historical comparator groups

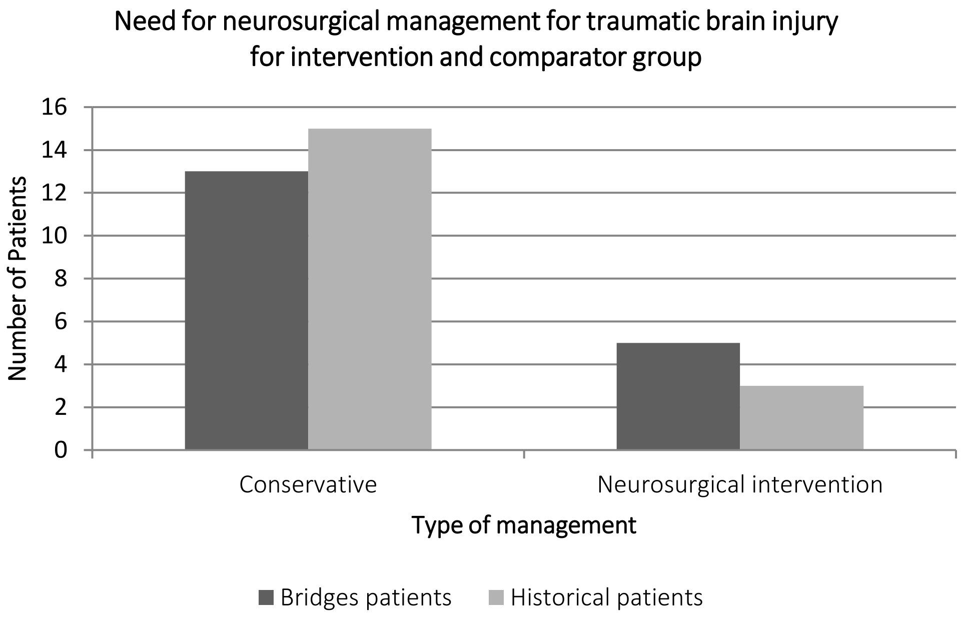

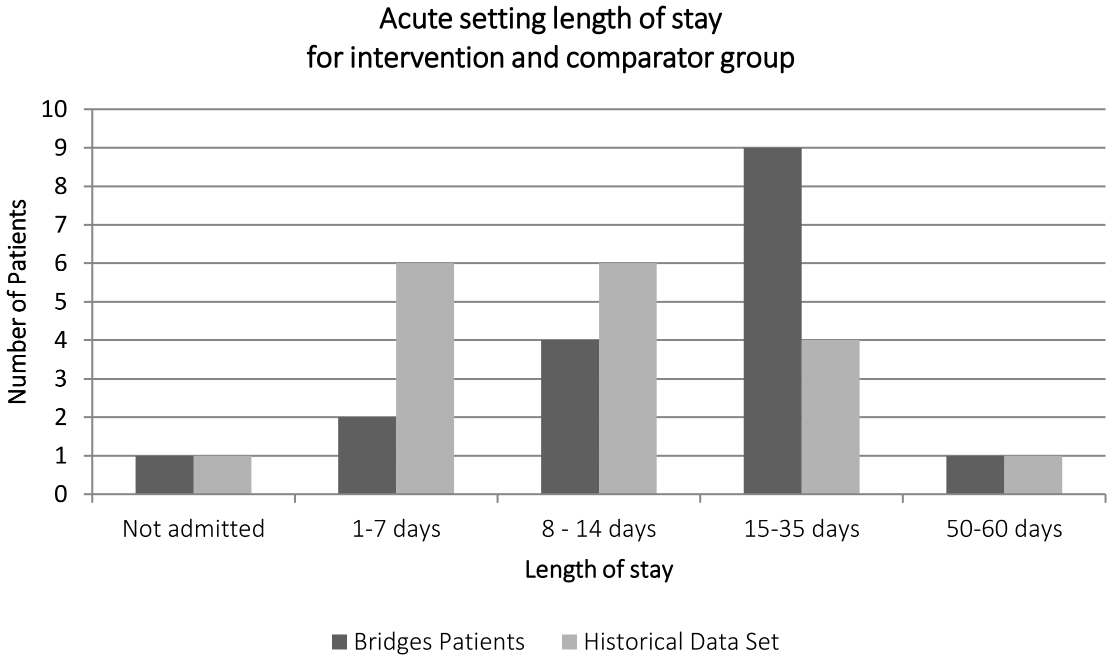

Appendix 4

A) Data for patient sample receiving intervention and comparator group: Hospital Anxiety and Depression Scale (HADS)

Hospital Anxiety and Depression Scale:
Anxiety Scores

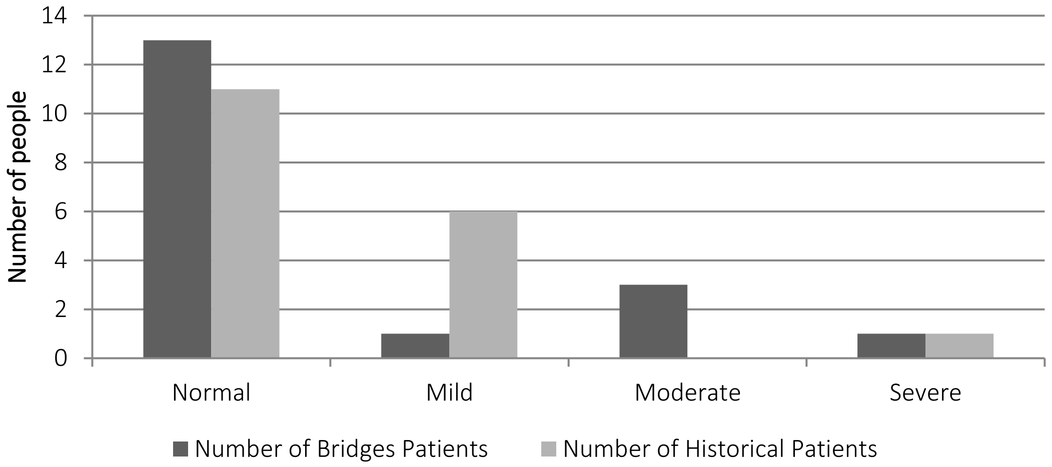

Hospital Anxiety and Depression Scale:
Depression Scores

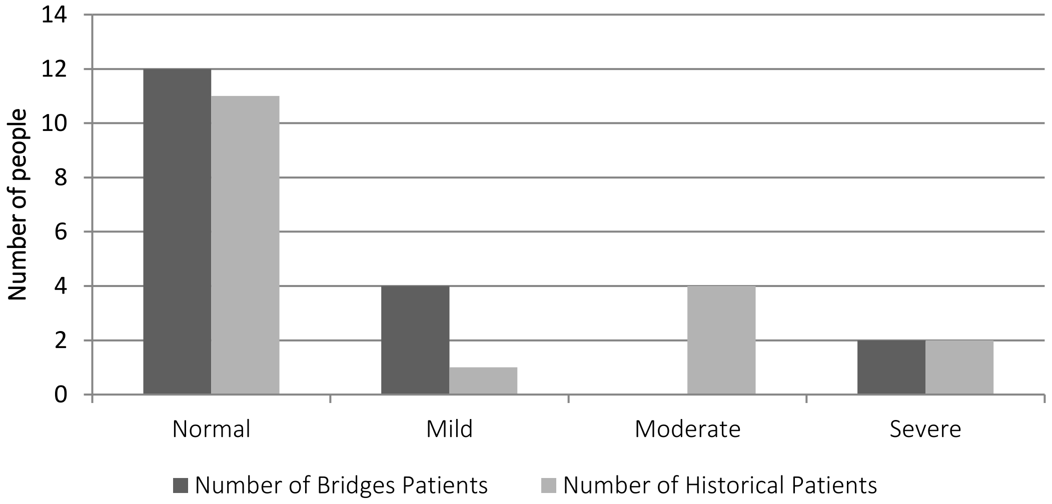

B) Data for patient sample receiving intervention and comparator group: Short Form Health Survey (SF36)

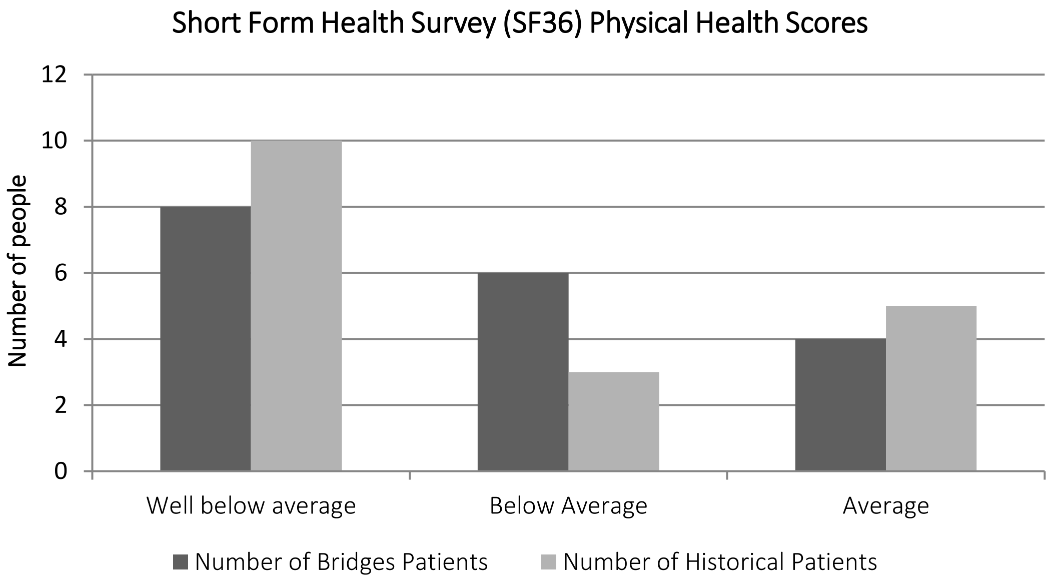

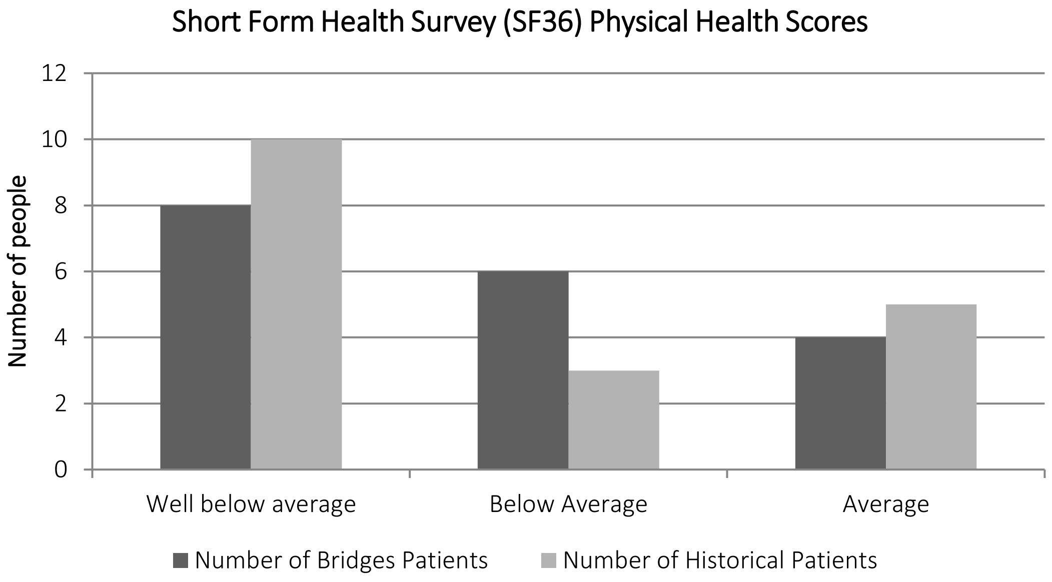

C) Data for sample of patients receiving intervention: Self Efficacy Scale (not available for comparator group)

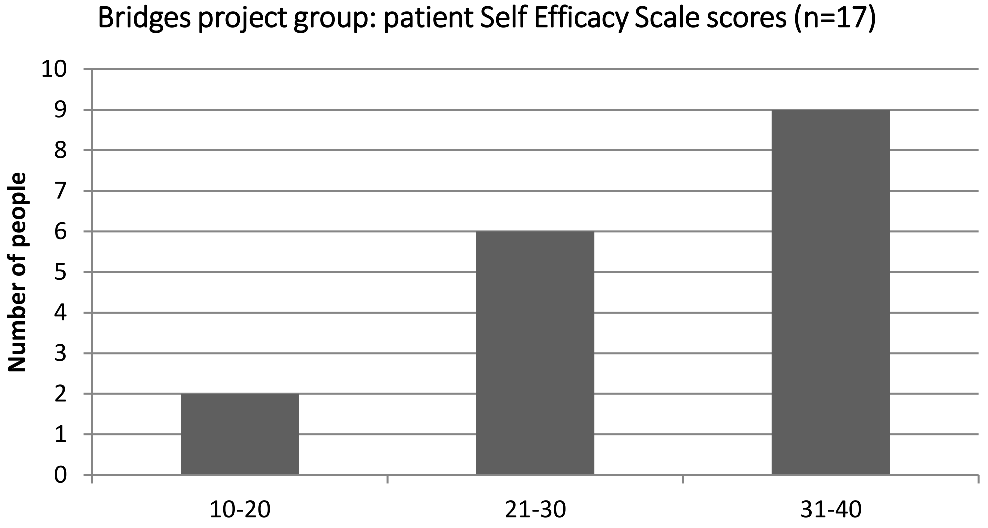

Scoring guide: the minimum possible score on this measure of self-efficacy is 10, with a maximum possible score of 40. A higher score indicates higher self-reported self-efficacy.
