# Supplementary material for: Supporting self‐management after traumatic brain injury: Codesign and evaluation of a new intervention across a trauma pathway
Source: Health Expect. 2019 Apr 29;22(4):632–42. doi: 10.1111/hex.12898 (PMC6737836; doi:10.1111/hex.12898)

Supplementary material
Sample pages from book used within supported self-management approach

Bridges
SELF-MANAGEMENT

Building bridges after brain injury

Contains stories, tips and ideas from people who have experienced a brain injury

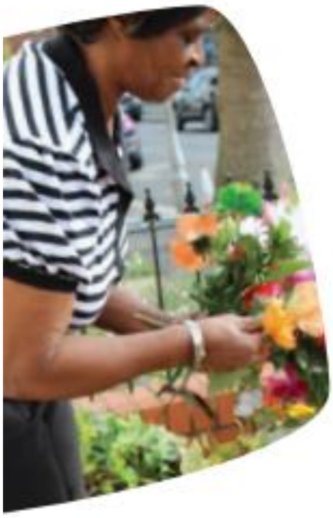

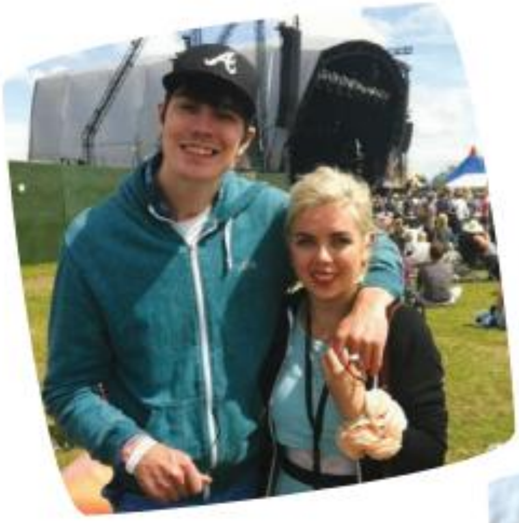

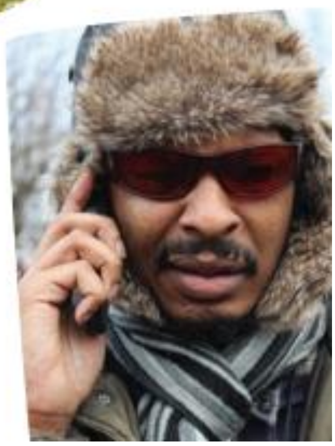

Supplementary material
Sample pages from book used within supported self-management approach

Contents

2 How to use the book
Guidance on how the book can best help you

12 People living with brain injury
The experiences of some other people after their brain injury

44 Managing my brain injury
Common issues experienced by people after brain injury and some of their own unique solutions

74 This is me
Activities which have helped

Bridges
SELF-MANAGEMENT

Supplementary material
Sample pages from book used within supported self-management approach

104 Reflection
Review and record
your progress so far

116 Planning for the future
Record some of your hopes

126 Taking control
Setting short term targets

142 Useful contacts

Supplementary material
Sample pages from book used within supported self-management approach

Common issues covered...

I can't remember my injury... See page 46

I even denied I had a brain injury... See page 48

Other people don't always understand... See page 52

Feeling alone... See page 54

Trouble remembering day-to-day things... See page 56

I react to things differently now... See page 58

No energy or Oomph... See page 60

I can feel overwhelmed... See page 62

I have felt worried, low and emotional... See page 64

Some relationships have changed... See page 66

I have had symptoms nobody told me about...
See page 68

I have to make adjustments and adapt... See page 70

Supplementary material
Sample pages from book used within supported self-management approach

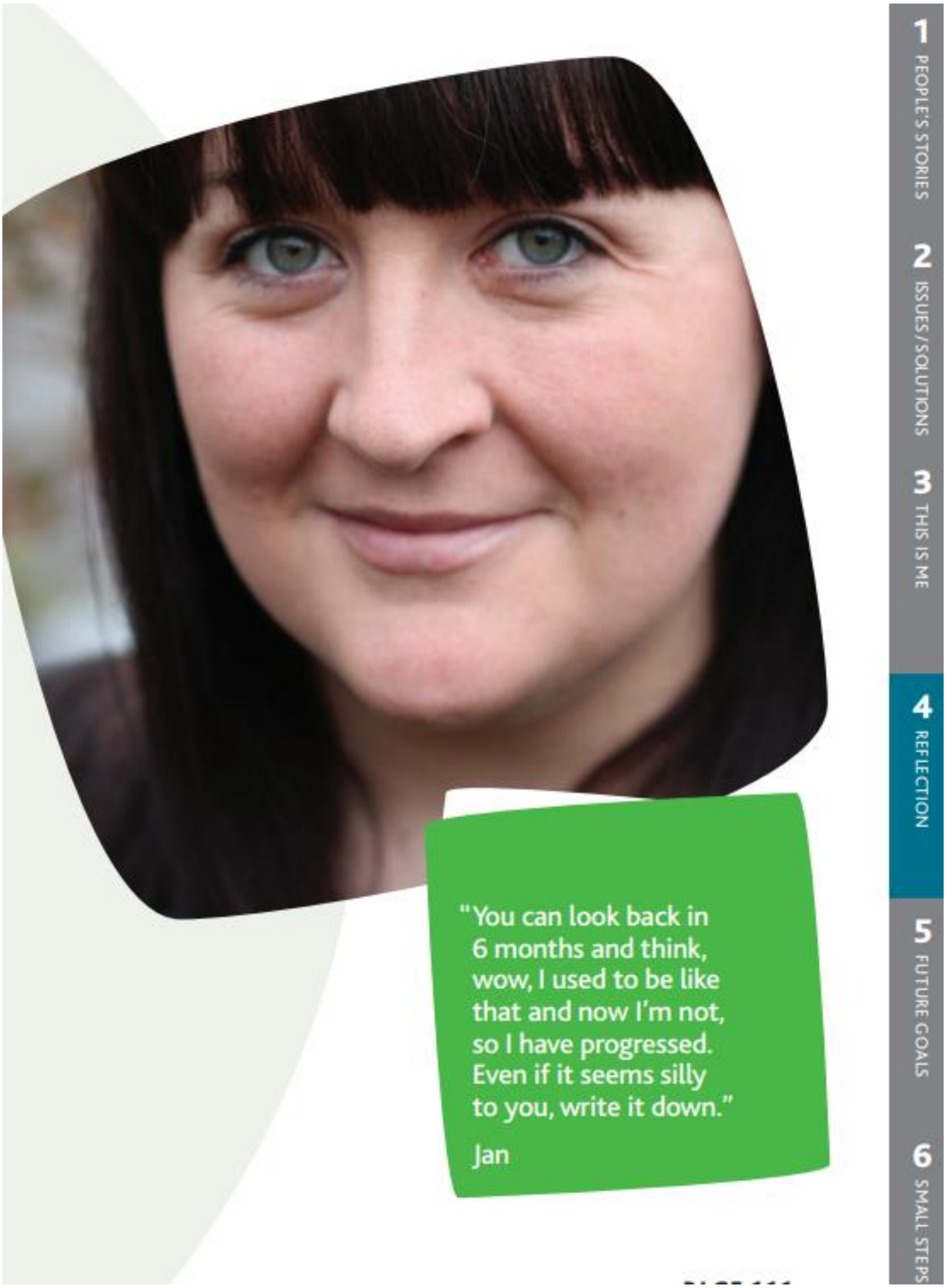

Supplement: Supplementary file 2 [file HEX-22-632-s002.pdf]
