# Supplementary material for: Supporting self‐management after traumatic brain injury: Codesign and evaluation of a new intervention across a trauma pathway
Source: Health Expect. 2019 Apr 29;22(4):632–42. doi: 10.1111/hex.12898 (PMC6737836; doi:10.1111/hex.12898)
Supplement: Supplementary file 3 [file HEX-22-632-s003.pdf]

Supplementary material
Sample pages from family and friends' book used within supported self-management approach

Bridges
SELF-MANAGEMENT

A book for family and friends of people with traumatic brain injury

Contains stories, ideas and tips from families and friends of people living with brain injury

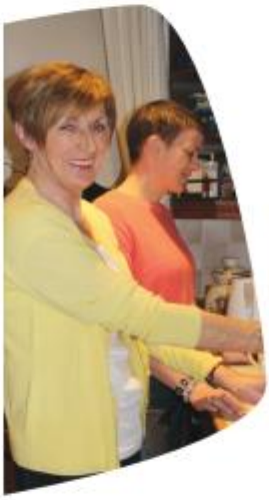

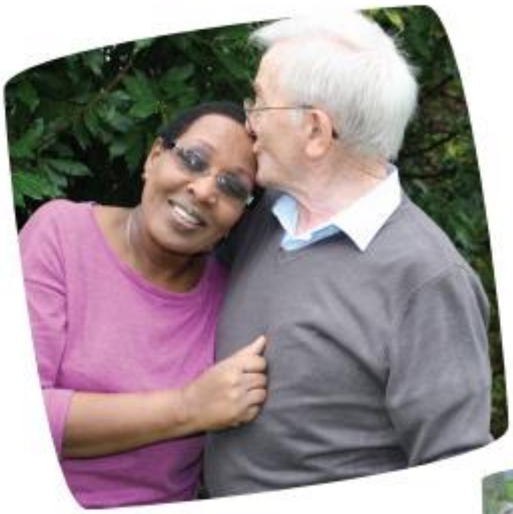

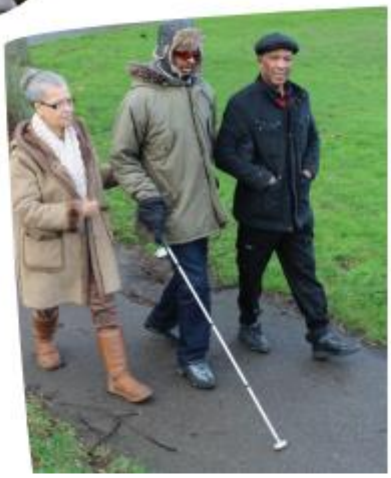

Supplementary material
Sample pages from family and friends' book used within supported self-management approach

Contents

01 Introduction

05 Family and friends of people with traumatic brain injury

22 Common issues and challenges

52 Using the 'Building Bridges' book with your family member or friend

60 Resources and contacts

Bridges
SELF-MANAGEMENT

Main issues

Dealing with news about the brain injury... See page 24

Not able to believe what has happened... See page 25

Feeling uncertain and helpless... See page 26

The ripple effect of brain injury... See page 28

Coping with hospital... See page 30

Feeling guilty, not taking care of yourself... See page 32

Finding a new rhythm, changes to my role/relationships... See page 34

Coping with the changes between hospital and home... See page 36

Looking for positives , keeping hopeful... See page 38

Involving other friends and family... See page 40

Some people not understanding... See page 42

Changes in your family member/friend... See page 44

Unexpected symptoms and challenges... See page 46

Working with healthcare staff... See page 48

Adjusting in the longer term... See page 50

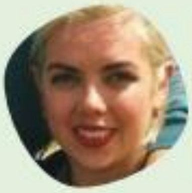

Ellen: 'I don't think anyone really prepared us for the emotional side and how different it is to live in the real world with it. In rehab it is very structured, like 10am this and then this. But then you have got to sort of make your own structure – it's sort of like, ok what is he going to do now, and are you going to sit at home all day eat biscuits? No.'

Mark: It was the lack of control when Sarah came home – what if she does go to the shop, falls over into the road and gets run over, or what if a tree falls on her? It's re-establishing that things are okay, and being able to take that step back. It's because it was so intense for that first month – I think we were hardly apart.
